# Feasibility and results of acute cardiac stress MRI with regadenoson for risk stratification of COPD-patients with NSTEMI

**DOI:** 10.1186/1532-429X-17-S1-P110

**Published:** 2015-02-03

**Authors:** Felix T Range, Florian Bönner, Britta Butzbach, Dirk Dinjus, Stefanie Keymel, Malte Kelm, Mirja Neizel-Wittke

**Affiliations:** Dept. of Cardiology, Pneumology and Angiology, University Hospital of Düsseldorf, Duesseldorf, Germany

## Background

Patients with chronic obstructive pulmonary disease (COPD) often present with symptoms equivalent to an acute coronary syndrome (ACS) and slightly elevated high sensitive troponine in the observational range (hsTnT 14-53 ng/l). While this cannot differentiate between coronary or pulmonary origin of myocardial damage it necessitates further investigation since it has prognostic impact. Comorbidity of COPD and coronary heart disease is frequent and risk factors are also congruent. In this acute setting, a safe, and reliable test to identify coronary problems is of great value. Non-invasive assessment of these patients is hampered by low echocardiographic imaging quality due to pulmonary emphysema and by the bronchospasmic effect of adenosine. With the new vasodilatator regadenoson, stress-MRI is possible in patients with COPD.

The aim of this study was to evaluate the value of regadenoson stress MRI in patients with known COPD and low troponine values.

## Methods

Ten patients (66±18 yo) with COPD GOLD grade I-III presenting with ACS and elevated hsTnT in the observational range (23±8 ng/l) were enrolled. Prior to coronary catheterization, they underwent cMRI with regadenoson-stress (400 µg i.v. bolus). Scans were conducted on a Philips Achieva 1.5 T MRI, including CINE-sequences, T2-imaging, late enhancement imaging, pulmonary angiography as well as rest and stress myocardial perfusion measurements. All Patients received pulmonary function testing before and after regadenoson and coronary angiography after cMRI.

## Results

All cMRI scans were conducted without any adverse effect. Pulmonary function testing revealed no deterioration after regadenoson (FEV1 pre and post MRI: 66% and 68%).

Quality of the scans remained unhampered by the emergency management. Regadenoson elevated heart rate and rate-pressure-product significantly by 29%. In three patients, stress cMRI detected myocardial ischemia. In all these patients, coronary catheterization revealed relevant coronary stenoses necessitating intervention in the respective vessel predicted by cMRI. All patients without myocardial ischemia in cMRI had no coronary stenosis in catheterization. Positive and negative predictive values of cMRI with regadenoson-stress were 100% in our population.

## Conclusions

Cardiac stress MRI proved safe in the acute setting of ACS management. Regadenoson did not affect pulmonary function. cMRI delivered reliable results with comparable quality to non emergency settings, showing positive and negative predictive values of 100% when determining the need for coronary revascularisation and predicting the target vessel.

This method could influence the future management of patients with COPD ACS and slightly elevated hsTnT bearing relative contraindications for coronary catheterization.

## Funding

None.

